# Synaptic abnormalities in a *Drosophila* model of Alzheimer’s disease

**DOI:** 10.1242/dmm.012104

**Published:** 2014-01-30

**Authors:** Siddhita D. Mhatre, Vivek Satyasi, Mark Killen, Brie E. Paddock, Robert D. Moir, Aleister J. Saunders, Daniel R. Marenda

**Affiliations:** 1 Department of Biology, Drexel University, Philadelphia, PA 19104, USA.; 2 Department of Biology, Arcadia University, Glenside, PA 19038, USA.; 3 Genetics and Aging Research Unit, MIND, Massachusetts General Hospital, Harvard Medical School, Boston, MA 02114, USA.; 4 Department of Biochemistry and Molecular Biology, Drexel University College of Medicine, Philadelphia, PA 19104, USA.; 5 Department of Neurobiology and Anatomy, Drexel University College of Medicine, Philadelphia, PA 19129, USA.

**Keywords:** APP, Alzheimer’s disease, *Drosophila*, BACE, Synapse, NMJ

## Abstract

Alzheimer’s disease (AD) is an age-related neurodegenerative disease characterized by memory loss and decreased synaptic function. Advances in transgenic animal models of AD have facilitated our understanding of this disorder, and have aided in the development, speed and efficiency of testing potential therapeutics. Recently, we have described the characterization of a novel model of AD in the fruit fly, *Drosophila melanogaster*, where we expressed the human AD-associated proteins APP and BACE in the central nervous system of the fly. Here we describe synaptic defects in the larval neuromuscular junction (NMJ) in this model. Our results indicate that expression of human APP and BACE at the larval NMJ leads to defective larval locomotion behavior, decreased presynaptic connections, altered mitochondrial localization in presynaptic motor neurons and decreased postsynaptic protein levels. Treating larvae expressing APP and BACE with the γ-secretase inhibitor L-685,458 suppresses the behavioral defects as well as the pre- and postsynaptic defects. We suggest that this model will be useful to assess and model the synaptic dysfunction normally associated with AD, and will also serve as a powerful *in vivo* tool for rapid testing of potential therapeutics for AD.

## INTRODUCTION

Alzheimer’s disease (AD) is a progressive, age-dependent and irreversible neurodegenerative disease that is currently the sixth leading cause of death in the US. It is the only cause of death within the top ten leading causes in the US that cannot be cured, prevented or even slowed [Alzheimer Association 2010 report: Changing the Trajectory of Alzheimer’s Disease: A National Imperative (http://www.alz.org/documents_custom/trajectory.pdf)]. AD is characterized by extensive loss of synaptic connections, neuronal death, and the presence of extracellular amyloid plaques and intracellular neurofibrillary tangles (iNFTs) (LaFerla and Oddo, 2005; Polidori et al., 2007). The iNFTs are dense intra-neuronal lesions composed of abnormally paired helical filaments (Selkoe, 2001), the main consitutent of which is hyperphosphorylated microtubule-associated tau protein (Grundke-Iqbal et al., 1986). The extracellular amlyoid plaques are mainly composed of the amyloid beta (Aβ) peptide. Aβ is a peptide formed by proteolytic processing of the amyloid precursor protein (APP) (Zhang et al., 2007). APP processing occurs by one of two main pathways: the non-amyloidogenic pathway, or the amyloidogenic pathway (Chow et al., 2010; Zhang et al., 2012). In the non-amyloidogenic pathway, α-secretase cleaves APP in the ectodomain within the Aβ region of the APP protein, which precludes the generation of the Aβ peptide (Chow et al., 2010; Zhang et al., 2012). In the amyloidogenic pathway, APP is cleaved initially by the β-site APP-cleaving enzyme (BACE), releasing a soluble APP fragment (sAPPβ) that is secreted outside the cell, leaving behind a membrane-associated C-terminal fragment of 99 or 89 amino acids [C99 or C89 (CTFβ)]. The CTFβ is then cleaved by γ-secretase, generating the Aβ peptide and a cytoplasmic APP intracellular domain (AICD) (Chow et al., 2010; Zhang et al., 2012). Aβ_42_ peptide oligomerizes, is neurotoxic (Iijima et al., 2004), and readily forms aggregates that accumulate in the brain to form plaques (Small et al., 2001). These oligomers are thought to cause inflammation, oxidative stress and apoptosis, thereby resulting in synaptic and neuronal loss (De Felice et al., 2007; Sakono and Zako, 2010; Tomiyama et al., 2010).

Literature suggests that the extracellular Aβ-containing plaques are associated with neuronal loss (Braak and Braak, 1998). Further evidence suggests that the loss of glutamatergic neurons in the hippocampus and cortex of AD patients could be an early event in AD pathogenesis (Revett et al., 2013). Glutamate is the major excitatory neurotransmitter present in the mammalian nervous system, and mediates processes that underlie learning and memory (Hu et al., 2007; Lüscher and Huber, 2010; Matsuo et al., 2008; Mattson, 2008; Walton and Dodd, 2007). In order to study the effects of Aβ on glutamatergic neuron function *in vivo*, we have used the *Drosophila* larva neuromuscular junction (NMJ) as a model system. The NMJ in the fly is a glutamatergic synapse, and is similar in composition and function to mammalian glutamatergic synapses in the central nervous system (Collins and DiAntonio, 2007). In total, 95.8% of all mammalian postsynaptic density (PSD) proteins have a homolog in the PSD of *Drosophila* NMJs (Liebl and Featherstone, 2008), making the fly NMJ a particularly tractable and powerful system for studying how synapses form and function (Collins and DiAntonio, 2007). It can be analyzed as a single glutamatergic synapse *in vivo*. Each presynaptic motor neuron and postsynaptic muscle cell is easily identifiable, and has stereotypical pre- and postsynaptic development that has been well characterized (Collins and DiAntonio, 2007).

Previously, we characterized a *Drosophila melanogaster* AD model (Chakraborty et al., 2011). This model was developed by expressing the human *APP_695_* and *BACE* genes within the central nervous system of *Drosophila*, and mimics many AD symptoms (Chakraborty et al., 2011). In this study, we have further investigated the effect of expressing the human *APP_695_* and *BACE* genes within glutamatergic motor neurons in the fly using two genetically independent fly lines. We observed that larvae expressing *APP* and *BACE* showed significantly reduced locomotor motion, altered synaptic morphology, and reduced muscle size at the larval muscle 6/7 NMJ. We also observed decreases in the total number of synaptic connections (boutons), and decreased mitochondrial intensity in motor neurons, as well as decreased postsynaptic markers in muscle cells. This is consistent with the synaptic loss observed in mammalian AD models. Each phenotype we observe in this model can be significantly suppressed by treating the larvae expressing APP and BACE with a γ-secretase inhibitor, L-685,458. We suggest that this model, accompanied with other studies, can allow us to better understand the synaptic defects associated with AD pathogenesis and can be efficiently used as an *in vivo* tool for testing potential AD therapeutics.

**TRANSLATIONAL IMPACT**

**Clinical issue**

Alzheimer’s disease (AD) is a progressive neurodegenerative disorder and is the most common cause of dementia in the developed world, affecting roughly 27-million people worldwide. There is currently no cure for AD, and the treatments that are available do not target the underlying mechanisms that cause the disease. Developing rapid, *in vivo*, animal models to test potential therapeutic intervention strategies for AD will be crucial to allow the future development of effective AD treatments.

**Results**

Here, the authors characterized the synaptic and behavioral deficits associated with a *Drosophila* model of AD. This study utilizes the *Drosophila* larval neuromuscular junction (NMJ) to investigate the effect of expressing the human AD-associated *APP* and *BACE* genes in the glutamatergic motor neurons of two genetically distinct fly lines with differential gene expression levels. Larvae expressing both human APP and BACE showed significant reductions in locomotion, reduced synaptic connections at the NMJ, and decreased mitochondrial localization in presynaptic motor neurons. Comparison of findings from the two fly lines revealed a dosage-dependent effect on the behaviors and morphology tested in this study. Furthermore, the authors demonstrated that feeding larvae expressing APP and BACE with a potent γ-secretase inhibitor (L-685,458) suppressed both the behavioral defects and the synaptic defects.

**Implications and future directions**

The findings reported in this study show that *Drosophila* expressing human APP and BACE demonstrate synaptic loss and behavioral deficits consistent with mammalian AD models. Pharmacological rescue of the observed defects showcase the utility of this NMJ-based model for rapid *in vivo* screening of potential drugs that could be used to treat AD in humans. Taken together, the study will help to better understand AD pathogenesis and aid its treatment.

## RESULTS

### Differential expression of APP in distinct genetic backgrounds

**Fig. 1.**
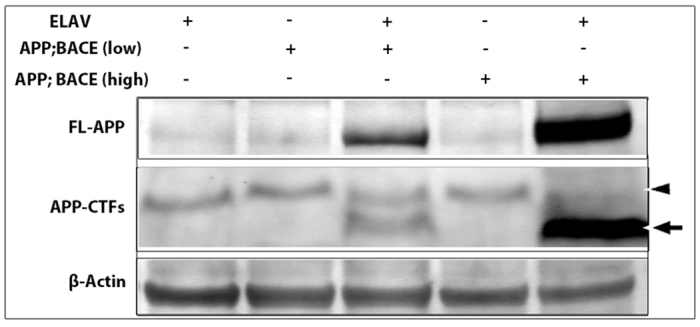
**Differential expression of transgenes in fly lines.** Western blot analysis of human APP and fly β-actin is detected in fly head lysates. Two independent fly lines were tested for the expression of the transgenes. Lane 1: *elav*; +; + heterozygous flies. Lane 2: +; *APP; BACE* (low) heterozygous flies. Lane 3: *elav; APP; BACE* (low) heterozygous flies. Lane 4: +; *APP, BACE*; + (high) heterozygous flies. Lane 5: *elav; APP, BACE*; + (high) heterozygous flies. FL-APP (full length APP; ~110 kD), APP-CTFs (C-terminal fragments; ~10–12 kD) and Appl-CTFs (~15 kD) were detected by C1/6.1. Arrow indicates β-CTFs in lanes 3 and 5. Arrowhead indicates Appl-CTFs in lanes 1–5. β-actin antibody was utilized for loading controls.

To express our transgenes we have utilized the bipartite Gal4/UAS system (Brand and Perrimon, 1993). We restricted the expression of the human *APP* and *BACE* transgenes to the central nervous system of the fly by using the *elav-Gal4* driver (Yao and White, 1994). In order to control for genetic background effects, we utilized two genetically independent fly lines. To confirm and compare the relative expression levels of our transgenes in these fly lines, we conducted western blot analysis. We detected full-length human APP in fly head lysates from both transgenic backgrounds when induced with *elav-Gal4* (Fig. 1). However, we also observed that expression of APP was significantly higher in one *APP; BACE* background compared with the other (compare lanes 3 with lanes 5, Fig. 1). We also observed the presence of BACE (supplementary material Fig. S1) and APPβ C-terminal fragments (CTFs), consistent with our previous work (Chakraborty et al., 2011), suggesting the proper expression and activity of the human β-secretase protease (Fig. 1, arrow). Again, we noted higher expression of these CTFs in one background compared with the other. Because of these observed differences in APP and CTF levels we referred to the two *APP; BACE* lines as APP; BACE (low) and APP; BACE (high).

In addition to exogenous human APP, we also noted the presence of what appeared to be endogenous fly Appl-CTFs generated through proteolysis of the fly homolog of APP (Appl) in all lanes. We confirmed that these bands were indeed the endogenous fly Appl protein through genetic overexpression of the Appl protein (lane 3 in supplementary material Fig. S1), and through analysis of protein levels in an *Appl* null mutant (lane 4 in supplementary material Fig. S1) (Groth et al., 2010; Rosen et al., 1989). Taken together, our data suggest that, although both *APP; BACE* genetic backgrounds are induced with the same *Gal4* driver, the relative levels of APP expression differ between them.

### Expression of human APP and BACE in larvae causes behavioral deficits

We have previously shown that expression of human APP and BACE in the fly central nervous system leads to decreased motor function in adult flies (Chakraborty et al., 2011). We next examined whether expression of human APP and BACE also shows motor deficits in developing larvae by analyzing larval contraction and crawling ability (Sinadinos et al., 2012). In *Drosophila* larvae, intact synaptic transmission from motor neurons results in coordinated peristaltic movement of the larval muscles causing crawling behavior of larvae. A full body wall contraction starts at the posterior end of the larvae, as the posterior body wall contracts, and it propagates in a wave towards the anterior end of the larvae, terminating on extension of the mouth hooks. Defects in larval locomotor behavior are often associated with neuronal and synaptic dysfunction (Folwell et al., 2010; Mudher et al., 2004).

**Fig. 2.**
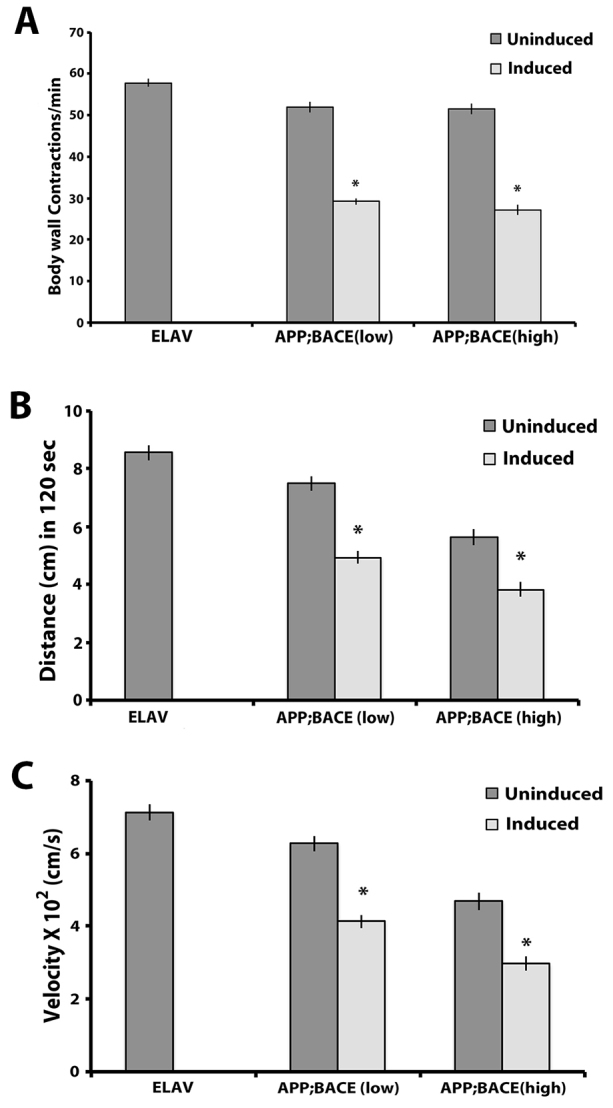
**Effect of APP and BACE expression on behavioral phenotypes of *Drosophila* third instar larvae.** (A) Number of body wall contractions per minute of third instar larvae under induced (*Gal4* with *UAS*) and uninduced (*Gal4* or *UAS* alone) conditions (*n*>30). (B) Distance crawled by larvae in 120 seconds (*n*>30). (C) Crawling velocity in cm/second for the third instar larvae under examination (*n*>30). Treatments are indicated. *P<0.05. Error bar represents standard error in each case.

We observed a significant decrease in larval body wall contractions in larvae expressing human APP and BACE from both genetic backgrounds compared with uninduced controls (Fig. 2A). We validated that these phenotypes were not due to expression of either human APP alone, or human BACE alone (supplementary material Fig. S2A), suggesting that the reduction in larval body wall contractions require the induction of both human APP and BACE together. We also observed a significant decrease in overall crawling distance in larvae expressing human APP and BACE (Fig. 2B), as well as a decrease in crawling rate (Fig. 2C) compared with uninduced controls. Although we did not observe a significant reduction in either crawling distance or crawling rate in flies that express human APP alone compared with uninduced controls (supplementary material Fig. S2B,C), we did observe a significant difference between flies expressing human BACE alone compared with uninduced controls (supplementary material Fig. S2B,C), suggesting that BACE expression alone is sufficient to observe motor behavior deficits. However, although expression of human BACE alone caused a 13% decrease in crawling distance and a 17% decrease in crawling rates, expression of both human APP and BACE caused a 34% decrease in crawling distance and a 36% decrease in crawling rates. Thus, expression of human BACE alone is not sufficient to explain the full motor defects observed for crawling distance and crawling velocity in larvae expressing both human APP and BACE together. Taken together, our data suggest that larvae expressing human APP and BACE in their central nervous system display defective motor behavior.

**Fig. 3.**
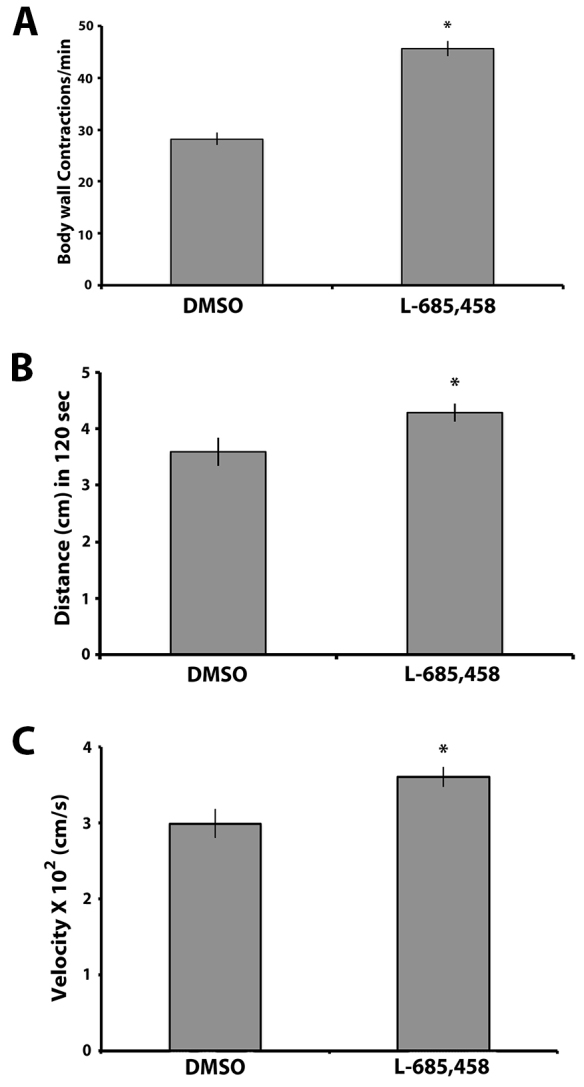
**γ-secretase inhibitor suppresses behavioral phenotypes in *Drosophila* third instar larvae expressing APP and BACE.** (A) Number of body wall contractions per minute of third instar larvae expressing APP; BACE (high) raised on food with either DMSO or L-685,458 (*n*>30). (B) Distance crawled by larvae in 120 seconds (*n*>30). (C) Crawling velocity in cm/second for the third instar larvae under examination (*n*>30). Treatments are indicated. *P<0.05. Error bar represents standard error in each case.

To validate that the phenotypes we observed are due to the production of Aβ in our model, we grew larvae expressing human APP; BACE (high) on food containing the γ-secretase inhibitor L-685,458. We observed that L-685,458 treatment led to a partial yet significant suppression of the contraction, crawling distance and crawling velocity deficits observed in this genetic background when compared with the larvae grown on the vehicle (DMSO) control food (Fig. 3A–C). This is consistent with our previous data showing that both Aβ_40_ and Aβ_42_ are produced in our model (Chakraborty et al., 2011). Taken together, these data suggest that the crawling deficits observed in our model are most likely due to the presence of Aβ peptides generated from functional endogenous γ-secretase activity.

### Expression of human APP and BACE in motor neurons alters synapse formation

**Fig. 4.**
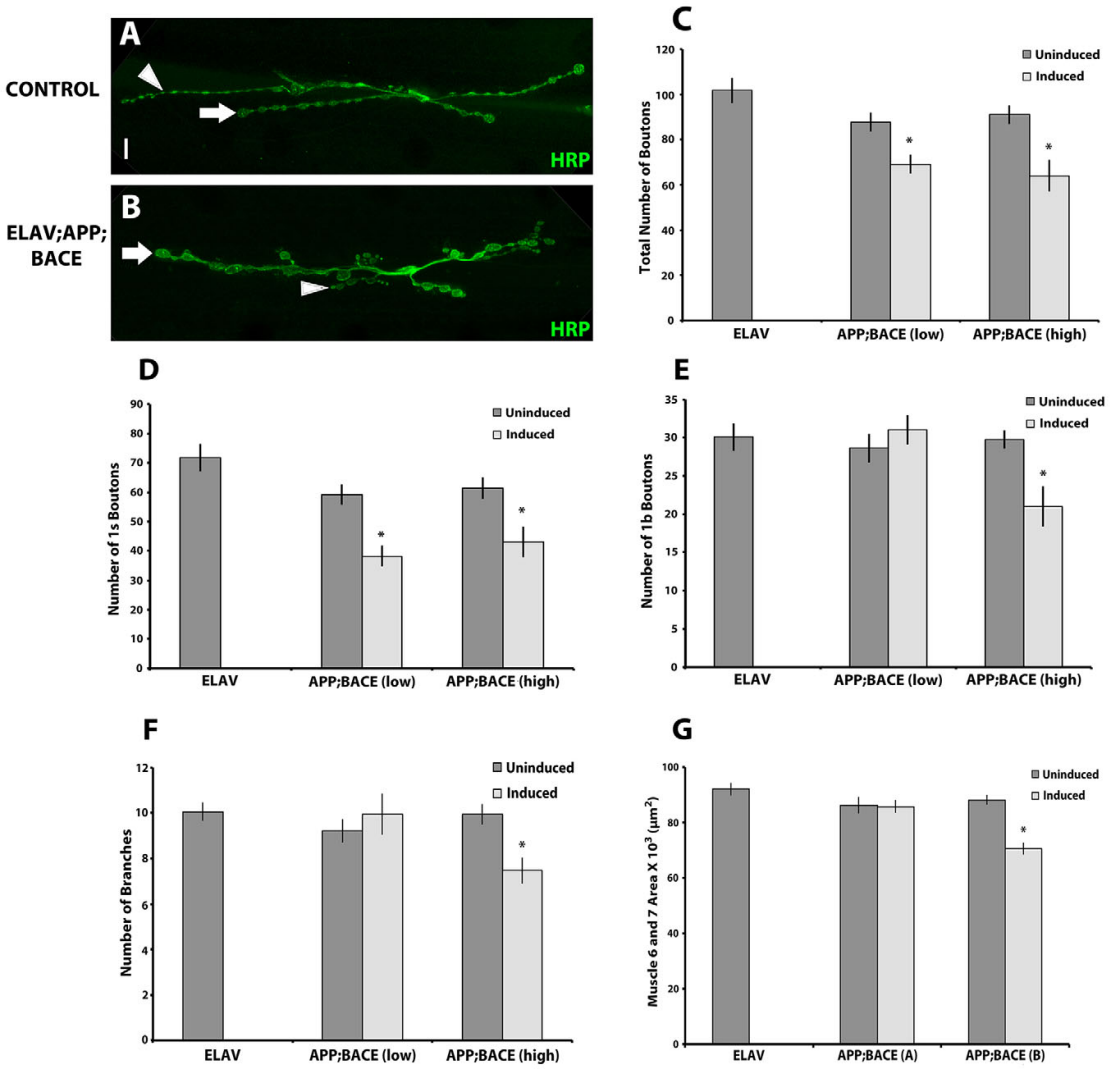
**Expression of human APP and BACE in *Drosophila* alters synapse formation.** (A,B) Confocal image of the synapse of segment A3, muscle 6/7 stained with neuronal marker, HRP. (A) *elav*; +; + heterozygous larvae. (B) *elav; APP; BACE* (low) heterozygous larvae. Scale bar: 10 μm. Arrows represent 1s type boutons; arrowheads represent 1b type boutons. (C–G) Histograms depict quantitative analysis of bouton and branch number on muscles 6 and 7 at abdominal segment 3. (C) Total number of boutons. (D) Number of 1s type boutons. (E) Number of 1b type boutons. (F) Total number of branches. (G) Average muscle 6 and 7 area. Treatments are indicated. Analysis represents *n*>15. *P<0.05. Error bar represents standard error in each case.

**Fig. 5.**
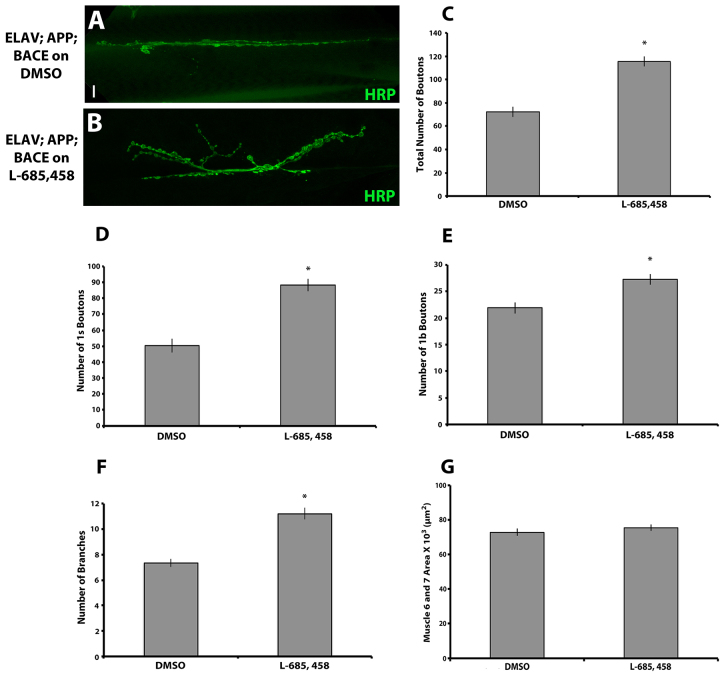
**Effect of γ-secretase inhibitor on synapse formation in *Drosophila* expressing APP and BACE.** Confocal image of the synapse of segment A3, muscle 6/7 stained with neuronal marker HRP. (A) *elav; APP; BACE* (high) heterozygous larvae fed on DMSO. (B) *elav; APP; BACE* (high) heterozygous larvae fed on L-685,458. Scale bar: 10 μm. (C–G) Histogram depicts quantitative analysis of bouton and branch number on muscles 6 and 7 at abdominal segment 3 on APP; BACE (high) heterozygous larvae fed on either DMSO or L-685,458. (C) Total number of boutons. (D) Number of 1s type boutons. (E) Number of 1b type boutons. (F) Total number of branches. (G) Average muscle 6 and 7 area. Treatments are indicated. Analysis represents *n*>15. *P<0.05. Error bar represents standard error in each case.

Based on the defective behavior in locomotion that we observe in animals expressing human APP and BACE, and because AD is a disease of synaptic dysfunction and loss, we next examined synapse formation of the motor neurons that innervate the larval body wall muscles. To analyze synapse formation in fly neurons that express human APP and BACE, we utilized muscles 6 and 7 of the NMJ of the third instar larvae in our AD models because this is a well-established model for studying synapse formation. First, we assessed the overall morphology of this synapse by confocal microscopy. This analysis revealed significant structural changes in this synapse of larvae expressing human APP and BACE compared with uninduced controls (Fig. 4A,B). Again, these structural changes could be suppressed by treating the larvae on food containing γ-secretase inhibitor L-685,458 (Fig. 5B).

Upon reaching the surface of the muscle fibers, the motor neurons that innervate the NMJ branch out and form a synaptic arbor composed of bead like structures (termed boutons) connected by thin axonal processes. We observed a significant reduction in total number of boutons in larvae expressing human APP and BACE as compared with both uninduced controls (Fig. 4C) and compared with larvae expressing either human APP alone or human BACE alone (supplementary material Fig. S3A). We also observed significant rescue of the total number of boutons upon treating larvae expressing human APP and BACE with L-685,458 as compared to larvae treated with the vehicle control DMSO (Fig. 5C).

*Drosophila* larval muscles 6 and 7 are innervated exclusively by type I boutons, which are further subdivided into type I small (1s) and type I big (1b) boutons (Atwood et al., 1997). 1s and 1b boutons are not only different in their structural properties, but differ in their functional properties as well. 1s boutons have larger amplitudes of excitatory junctional currents (EJCs) and stimulation thresholds are greater than type 1b boutons (Atwood et al., 1997; Koh et al., 2000). We observed that larvae expressing human APP and BACE showed ~35% reduction in the number of 1s boutons (Fig. 4D) in both the genetic backgrounds tested. Furthermore, the APP; BACE (high) background also showed a significant reduction in 1b boutons (Fig. 4E) most likely due to higher expression of human APP and BACE in this genetic background (Fig. 1). Control genetic backgrounds expressing human APP alone or human BACE alone had no significant effect on either 1s or 1b boutons count (supplementary material Fig. S3B,C).

The total area of the motor neuron innervation of the NMJ was not significantly reduced in larvae expressing human APP and BACE from either genetic background compared with controls (data not shown). However, the APP; BACE (high) model showed a significant decrease in the amount of branching in the motor neuron (Fig. 4F), as well as significantly reduced size of muscles 6 and 7 at the NMJ (Fig. 4G) compared with both uninduced controls, and compared with larvae expressing either human APP alone or human BACE alone (supplementary material Fig. S3D,E).

We next tested whether feeding larvae expressing human APP; BACE (high) on L-685,458 could suppress the structural defects we observe at this synapse. We observed that larvae treated with L-685,458 showed a significant increase in 1s bouton number (Fig. 5D) as well as a partial yet significant increase in 1b bouton number (Fig. 5E) as compared with the larvae cultured on DMSO vehicle food. We also observed a significant increase in the number of motor neuron branches at muscle 6 and 7 in larvae treated with L-685,458 as compared with DMSO (Fig. 5F). Although, the γ-secretase inhibitor suppressed most of the structural defects associated with the neurons, the reduction in the size of muscle 6 and 7 was not rescued with L-685,458 (Fig. 5G).

Taken together, these results suggest that the high expression of human APP and BACE in the larval motor neurons leads to a reduction in the connectivity and innervation of these neurons for their target muscle. This reduced connectivity can be suppressed by treating the larvae with γ-secretase inhibitor.

### Expression of human APP and BACE in motor neurons affects both pre- and postsynaptic development

A synapse is organized into a presynaptic terminal, where the neurotransmitter release machinery and synaptic vesicle pool is present, and PSD, where neurotransmitter receptors and ion channels are present. Because of the significant effects that expression of human APP and BACE had on the morphology of the presynaptic motor neuron, we next determined whether the expression of human APP and BACE affected molecular aspects of motor neuron development. To address this question, we analyzed the distribution and presence of two presynaptic proteins that are vital for proper synaptic functioning: Bruchpilot (Brp) and Cysteine string protein (CSP). Brp shows homology to the mammalian active zone protein ELKS/CAST (Kittel et al., 2006; Wagh et al., 2006), and is specifically localized to the presynaptic release sites (termed active zones) where synaptic vesicles fuse to the presynaptic membrane. Lack of Brp leads to mislocalization of Ca^2+^ channels, causing improper active zone maturation (Fouquet et al., 2009; Kittel et al., 2006; Wagh et al., 2006).

**Fig. 6.**
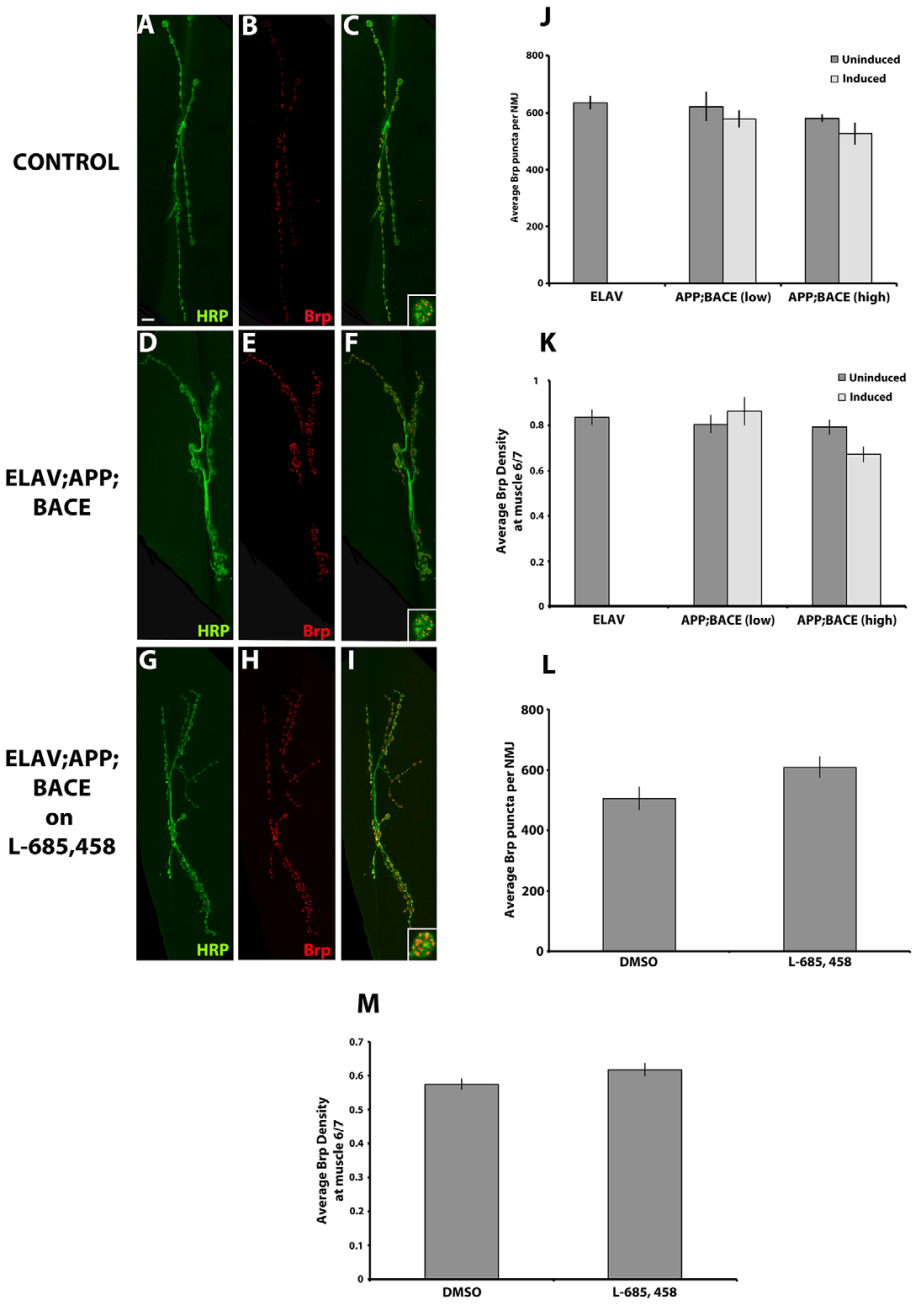
**Expression of human APP and BACE in *Drosophila* does not significantly alter active zones in synapses.** (A–I) Confocal images of the synapse of segment A3, muscle 6/7 stained with neuronal marker HRP (green) and active zone marker Brp (red). (A–C) *elav*; +; + heterozygous larvae. (D–F) *elav; APP; BACE* (low) heterozygous larvae. (G–I) *elav; APP; BACE* (high) heterozygous larvae fed on L-685,458. Color merges are shown (C,F,I) with higher magnification of one bouton in the lower right. Scale bar: 10 μm. (J,K) Histograms depict quantitative analysis on larvae fed on food without drug. (J) Average number of active zones per NMJ. (K) Average Brp density. (L,M) Histograms depict quantitative analysis on *elav; APP; BACE* (high) heterozygous third instar *Drosophila* larvae fed on either DMSO or L-685,458. (L) Average number of active zones per NMJ. (M) Average Brp density. Treatments are indicated. Analysis represents *n*=8–10. *P<0.05. Error bar represents standard error in each case.

We observed no significant difference in the total number of Brp puncta per NMJ from larvae expressing both human APP and BACE compared with both uninduced controls (Fig. 6J), and compared with larvae expressing either human APP alone or human BACE alone (supplementary material Fig. S4A), although a trend was noted for each genetic background (Fig. 6J). Quantification of active zone density for synaptic innervation at muscle 6 and 7 also showed no significant difference between larvae expressing human APP and BACE when compared with uninduced controls, APP alone, and BACE alone genetic backgrounds (Fig. 6K and supplementary material Fig. S4B). Furthermore, feeding larvae on γ-secretase inhibitor food did not show any significant effect compared with the vehicle control (Fig. 6L,M). Taken together, our data suggest that, although there is a significant loss of boutons overall in larvae expressing human APP and BACE compared with controls, there is no significant loss of Brp puncta or Brp puncta density per bouton in the boutons that do remain.

**Fig. 7.**
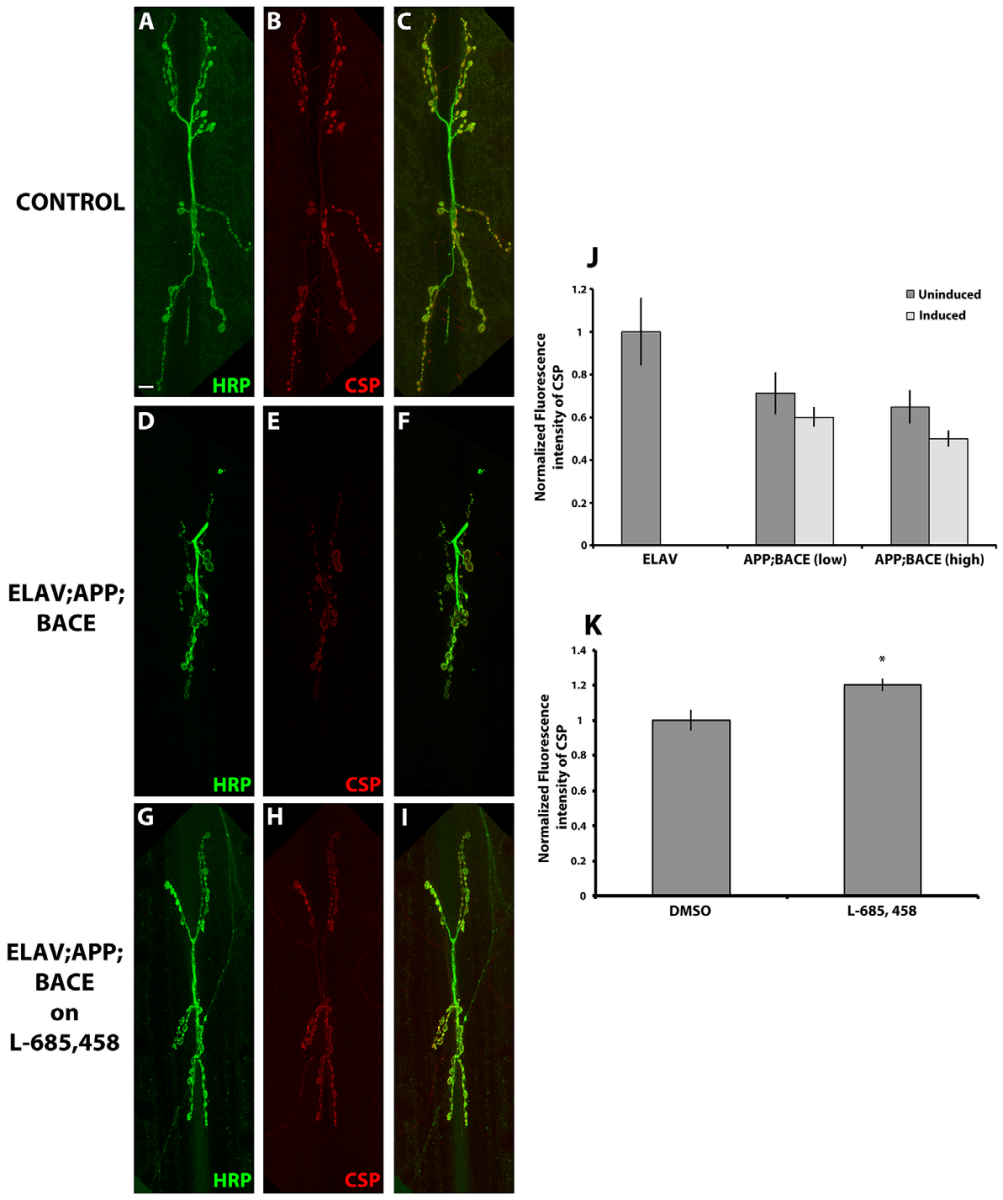
**Expression of human APP and BACE in *Drosophila* does not alter synaptic vesicular protein CSP.** (A–I) Confocal image of the synapse of segment A3, muscle 6/7 stained with neuronal marker, HRP (green) and presynaptic vesicle protein, CSP (red). (A–C) *elav*; +; + heterozygous larvae. (D–F) *elav; APP; BACE* (low) heterozygous larvae. (G–I) *elav; APP; BACE* (high) heterozygous larvae fed on L-685,458. Color merges are shown in C,F,I. Scale bar: 10 μm. (J,K) Histograms depict quantitative analysis. (J) Normalized fluorescence intensity of CSP. (K) Fluorescence intensity of CSP for *elav; APP; BACE* (high) heterozygous larvae fed on either DMSO or L-685,458 normalized to the vehicle control (DMSO). Treatments are indicated. Analysis represents *n*=10–14. *P<0.05. Error bar represents standard error in each case.

We also analyzed the expression of the cysteine string protein (CSP). CSP is evolutionarily conserved from invertebrates to humans (Zinsmaier et al., 1990), and is associated with membranes of synaptic and secretory vesicles (Dawson-Scully et al., 2007). It is a required protein for synaptic growth and to prevent neurodegeneration (Fernández-Chacón et al., 2004). We did not observe a significant difference in CSP intensity at presynaptic terminals in larvae expressing human APP and BACE compared with uninduced controls (Fig. 7J), or compared with controls expressing APP and BACE alone (supplementary material Fig. S4C), although we did observe a trend in both genetic backgrounds. Interestingly, the trend for decreased CSP intensity might be caused by expression of APP alone (supplementary material Fig. S4C). Again, the trend observed in CSP intensity upon expressing human APP and BACE could be significantly suppressed by feeding these larvae the γ-secretase inhibitor L-685,458 (Fig. 7K).

**Fig. 8.**
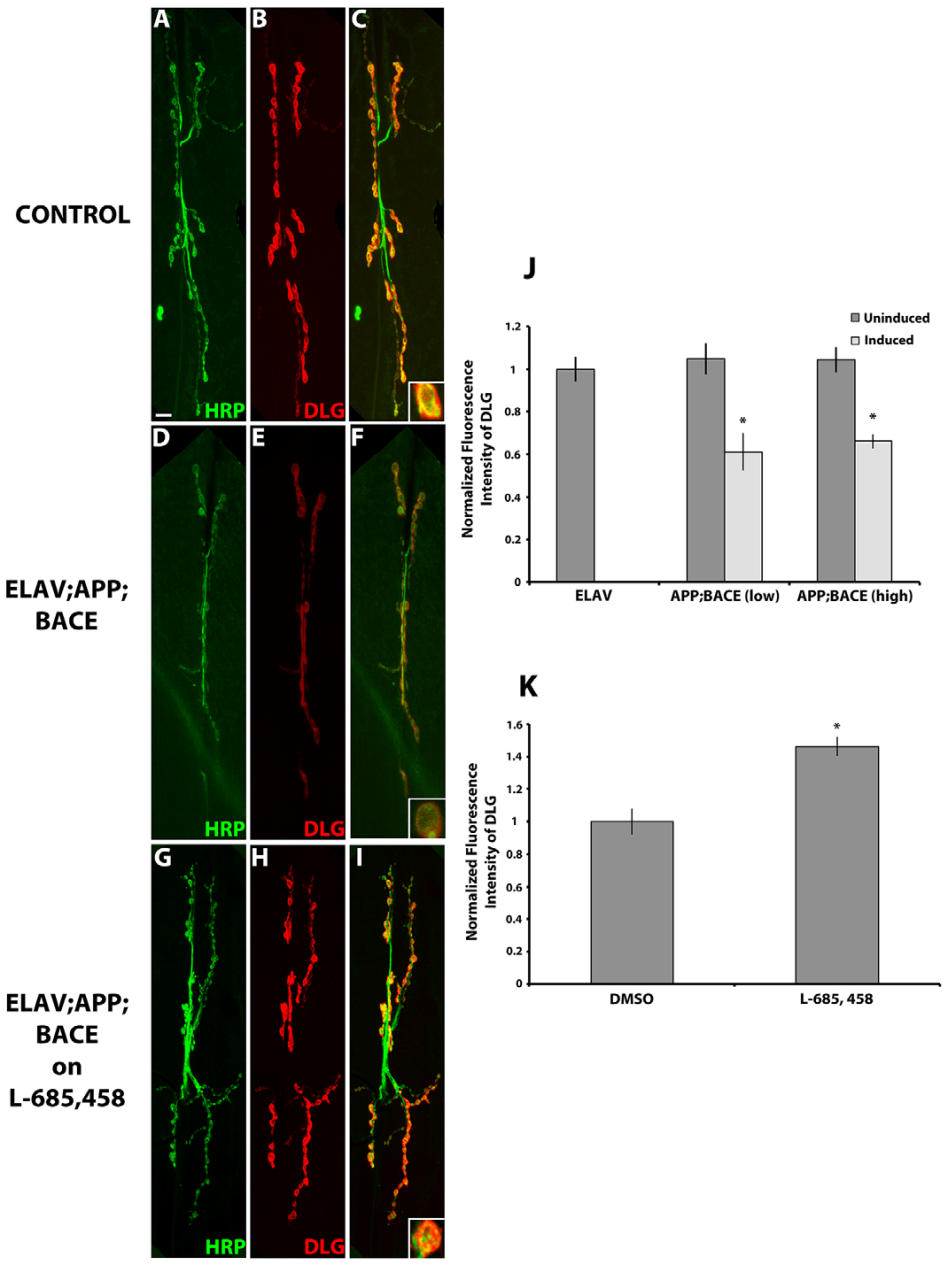
**Expression of human APP and BACE in *Drosophila* alters the postsynaptic protein DLG.** (A–I) Confocal image of the synapse of segment A3, muscle 6/7 stained with neuronal marker HRP (green) and postsynaptic protein DLG (red). (A–C) *elav*; +; + heterozygous larvae. (D–F) *elav; APP; BACE* (low) heterozygous larvae. (G–I) *elav; APP; BACE* (high) heterozygous larvae fed on L-685,458. Color merges are shown (C,F,I) with higher magnification of one bouton in the lower right. Scale bar: 10 μm. (J,K) Histograms depict quantitative analysis. (J) Normalized fluorescence intensity of DLG. (K) Fluorescence intensity of DLG for *elav; APP; BACE* (high) heterozygous larvae fed on either DMSO or L-685,458 normalized to the vehicle control (DMSO). Treatments are indicated. Analysis represents *n*=10–14. *P<0.05. Error bar represents standard error in each case.

We next examined expression of the *Drosophila* homolog of PSD-95, discs-large (DLG), which belongs to the membrane-associated guanylate kinases (MAGUKs) class of mammalian proteins (Thomas et al., 2000). In *Drosophila*, the presence of DLG at the postsynaptic specialization is crucial for the abundance of synaptic glutamate receptors (Chen and Featherstone, 2005). We observed a significant reduction in DLG protein levels in larvae expressing human APP and BACE in both genetic backgrounds compared with uninduced controls (Fig. 8J), and compared with larvae expressing APP and BACE alone (supplementary material Fig. S4D). Growing larvae expressing human APP; BACE (high) on L-685,458 significantly suppressed the effects of *APP; BACE* expression on DLG fluorescent levels at the synapse (Fig. 8K). These data suggest that expression of human APP and BACE in the presynaptic motor neurons can significantly affect the expression and/or localization of postsynaptic machinery in the muscle cell at the NMJ.

### Expression of human APP and BACE in motor neurons affects mitochondria

**Fig. 9.**
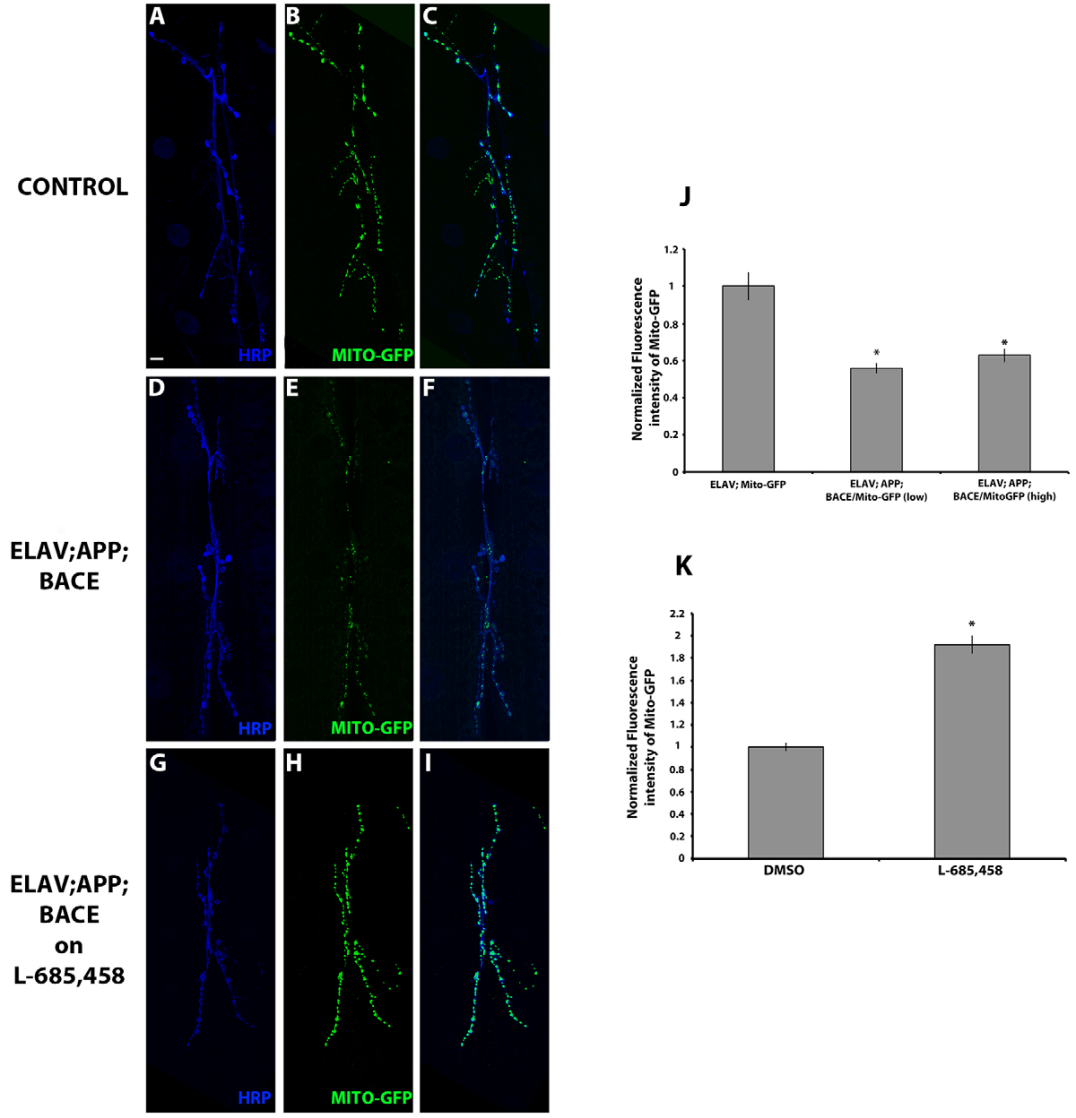
**Expression of human APP and BACE in *Drosophila* alters mitochondrial localization in the motor neurons.** (A–I) Confocal images of the synapse of segment A3, muscle 6/7 stained with neuronal marker HRP (blue) and Mito-GFP (green). (A–C) *elav*; +; *Mito-GFP* heterozygous larvae. (D–F) *elav; APP; Mito-GFP/BACE* (low) heterozygous larvae. (G–I) *elav; APP; Mito-GFP/BACE* (high) heterozygous larvae fed on L-685,458. Color merges are shown (C,F,I). Scale bar: 10 μm. (J,K) Histograms depict quantitative analysis. (J) Normalized fluorescence intensity of Mito-GFP. (K) Fluorescence intensity of Mito-GFP for *elav; APP; BACE* (high) heterozygous larvae fed on either DMSO or L-685, 458 normalized to the vehicle control (DMSO). Treatments are indicated. Analysis represents *n*=8–15. *P<0.05. Error bar represents standard error in each case.

Mitochondria are dynamic organelles whose active movement in the cell body is essential for calcium signaling, energy transfer and distribution in cells such as neurons (Chang et al., 2011; Szabadkai and Duchen, 2008; Yi et al., 2004). Previous literature suggests that Aβ_42_ induces mitochondrial mislocalization, which leads to mitochondrial dysfunction (Iijima-Ando et al., 2009), and various studies on human AD postmortem brains (Devi et al., 2006; Manczak et al., 2004; Smith et al., 1996) and mouse AD models (Du et al., 2010; Lee et al., 2012b; Trushina et al., 2012), as well as in *Drosophila* models, suggest that alteration of mitochondrial dynamics precedes AD pathophysiology (Iijima-Ando et al., 2009; Iijima-Ando et al., 2012). Therefore, we utilized a Mito-GFP reporter fly line that targets GFP to the mitochondrial matrix (Pilling et al., 2006) to assess mitochondrial abundance in flies expressing human APP and BACE. Consistent with previous studies, we observed a significant reduction in mitochondrial intensity levels in flies expressing human APP and BACE compared with uninduced controls (Fig. 9J), and compared with larvae expressing APP and BACE alone (supplementary material Fig. S5). Again, feeding larvae expressing APP; BACE (high) on γ-secretase inhibitor L-685,458 suppressed the reduction in mitochondrial intensity levels (Fig. 9K). Taken together, our results further confirm that expression of Aβ at the synapse can affect mitochondrial localization.

## DISCUSSION

AD is an age-related disease associated with loss of synapses, synaptic function and neurons, and mitochondrial abnormalities. In order to model disease pathogenesis and identify molecules that could prevent AD progression, animal AD models have proved essential. To maximize the utility of these models, they should be able to recapitulate symptoms and behaviors that are seen in AD patients.

*Drosophila* has emerged as an excellent invertebrate model system for studying human neurodegenerative diseases like AD (Bonini and Fortini, 2003; Chakraborty et al., 2011; Gama Sosa et al., 2012; Jackson et al., 1998; Mhatre et al., 2013; Watson et al., 2008). Previously, our lab has successfully developed and characterized an adult fly model for AD (Chakraborty et al., 2011). This model displays symptoms similar to clinical AD patients, including increased Aβ production, Aβ puncta in brains, decreased neuroanatomical areas associated with learning and memory, and defective memory (Chakraborty et al., 2011). We also showed that the disease phenotypes displayed by this model could be rescued pharmacologically with a γ-secretase inhibitor (Chakraborty et al., 2011). Because AD is a disease of synaptic loss and dysfunction, we have analyzed the morphology and development of the glutamatergic larval NMJ to determine how this synapse was affected in this model.

The *Drosophila* NMJ is an experimentally accessible, physiologically well-characterized and morphologically simple model system (Collins and DiAntonio, 2007; Schuster, 2006). Like excitatory synapses in the vertebrate central nervous system, the *Drosophila* NMJ relies on signaling by glutamate (Koh et al., 2000). In the study described here, we have expressed human *APP* and *BACE* genes in two separate genetic backgrounds, in fly post-mitotic glutamatergic motor neurons, allowing the normal proteolytic processing of APP to produce Aβ. Our results indicate that both genetic backgrounds successfully express and proteolytically cleave APP to yield APP CTFs (Fig. 1). Our previous studies have shown that this model successfully produces Aβ_42_ peptides (Chakraborty et al., 2011), and would therefore be of utility for rapid screening of drug efficacy and toxicity *in vivo*. The studies that we present here expand upon the utility of this model. For example, third instar *Drosophila* larvae exhibit peristaltic patterns of motion for locomotor behavior. This movement is highly rhythmic, stereotypic and coordinated, and is regulated by coordinated action of neural circuits including motor neurons, sensory feedback neurons and interneurons (Kohsaka et al., 2012). Electrophysiological data have displayed rhythmic recording of activity of motor neurons occurring concomitantly with a wave of contraction of fly larvae (Fox et al., 2006). The larvae expressing human APP and BACE in our study showed behavioral deficits in body wall contraction and crawling, suggesting defects in neural circuitry underlying this larval locomotion. We have further shown that the behavioral deficit seen in these AD larvae can be significantly suppressed by culturing the larvae on L-685,458, a γ-secretase inhibitor. The γ-secretase inhibitor precludes the formation of toxic Aβ_42_ peptide, thus demonstrating that the locomotion defect can be used for fast and efficient screening of potential target genes and pharmacological agents capable of targeting the γ-secretase complex.

Previous studies in cultured neurons and invertebrate and vertebrate animal models suggest that application or expression of Aβ_42_ in neurons results in reduced dendritic spines, decreases in synaptic protein levels and loss of memory (Borlikova et al., 2013; Jacobsen et al., 2006; Ripoli et al., 2013; Zempel and Mandelkow, 2012; Zhao et al., 2010). Recently, Sarantseva et al. expressed human APP and BACE in motor neurons using the *D42-Gal4* driver (Sarantseva et al., 2012). Consistent with our observations, these authors found decreased mitochondrial intensity in these motor neurons (Sarantseva et al., 2012). However, these authors described an overall increase in total bouton number, including increased 1s and 1b boutons, as well as an increase in branching at this synapse (Sarantseva et al., 2012). This is in direct contrast to what we have observed in the two distinct genetic backgrounds we assayed in this work.

What could account for these differences? Although both our group and Saratseva et al. restricted expression of the APP and BACE proteins to the presynaptic motor neurons, we utilized different *Gal4* drivers to do so. Sarantseva et al. utilized the *D42-Gal4* driver (Sarantseva et al., 2012), whereas we utilized the *Elav-Gal4* driver. Thus, expression of the *APP* and *BACE* transgenes in our model might be higher than that expressed in the Sarantseva et al. study. Indeed, we observed a dose-dependent difference in phenotypes between our two backgrounds, with the APP; BACE (low) background occasionally showing weaker effects than the APP; BACE (high) background, suggesting that the levels of APP expression matter significantly for phenotypic outcome. Furthermore, Sarantseva et al. used muscle 4 of segment 3, whereas we utilized muscle 6/7 of segment 3 in our studies. Thus, differences in bouton number might also be explained by the different specific motor neuron innervations analyzed.

Previous work has been performed on the *Drosophila* homolog for human APP, Appl (Rosen et al., 1989), on the NMJ. This work shows that overexpression of Appl in the motor neurons leads to disrupted axonal transport (Torroja et al., 1999a) and an increased number of boutons (Torroja et al., 1999b). Furthermore, the increase in synapse formation is due to signaling between FasII, Appl and Appl binding protein X11/Mint (Ashley et al., 2005). Expression of human APP and BACE in our model resulted in significant decreases in bouton number at the NMJ. We suggest that this is most likely because of the presence of high levels of Aβ_42_ within our model (Chakraborty et al., 2011), which might not be present when Appl is overexpressed alone.

Previously it was shown that expression of Aβ_42_ within motor neurons leads to a reduction in neurotransmitter release and decreased synaptic signaling (Chiang et al., 2009), consistent with our observations that expression of human APP and BACE in fly motor neurons leads to decreased larval locomotion. Further, previous literature has shown that expression of Aβ_42_ itself at the larval NMJ leads to a decrease in bouton number (Lee et al., 2012a).

We observed a significant decrease in DLG fluorescence in flies expressing human APP and BACE compared with controls. DLG belongs to family of PSD MAGUK proteins (Budnik et al., 1996). MAGUKs are required for the recruitment and stabilization of many synaptic proteins including glutamate receptors in the PSD (Fanning and Anderson, 1999; Sheng and Pak, 1999). Proper presynaptic innervation is required for clustering of DLG at the post-synapse and then localization of glutamate receptors at the PSD follows (Chen and Featherstone, 2005). Consistent with these findings, we observed a decrease in both presynaptic innervation and DLG fluorescence levels upon expression of APP and BACE in the nervous system. *dlg* mutant larvae display selective loss of glutamate receptors at the NMJ (Chen and Featherstone, 2005), and DLG protein is required for synaptic plasticity (Budnik et al., 1996; Thomas et al., 1997). We suggest that decreased DLG levels in our model are due to decreased signaling from the presynaptic motor neuron, and might lead to decreased glutamate receptors and reduced synaptic function.

In summary, we report here the effects of expression of the human APP and BACE proteins in presynaptic motor neurons at the *Drosophila* NMJ. We observe a significant effect on the development and morphology of this NMJ, which correlates well with behavioral deficits observed in larvae expressing human APP and BACE. Finally, we observe strong pharmacological rescue of the phenotypes upon feeding larvae expressing human APP and human BACE with L-685,458, suggesting that this model is amenable to identifying potential pharmacological agents that can be further tested for AD. Taken together, we suggest that this is another aspect of our model that can be utilized for rapid screening of potential target genes and therapeutics for AD.

## MATERIALS AND METHODS

### *Drosophila* stocks and genetics

All fly stocks and crosses were maintained at 25°C in a 12:12 light:dark cycle at 60% humidity unless otherwise indicated. All crosses were carried out at 25°C. Normal food consisted of a standard cornmeal, yeast and molasses recipe (Chakraborty et al., 2011). BL# refers to Bloomington Stock Center stock number (http://flystocks.bio.indiana.edu/bloomhome.htm). The GAL4/UAS system was used for the overexpression of UAS transgenes in *Drosophila* as described (Brand and Perrimon, 1993). Bloomington stock *P{GawB}elav^C155^* (BL#458) was used to drive transgene expression in the nervous system and are abbreviated in the text as elav. The *P{UAS:APP_695_}; P{UAS:BACE}* and *P{UAS:APP_695_}* (Greeve et al., 2004) stock, referred to in the text as APP; BACE (low) and APP alone, respectively, were generous gifts from Rita Reifegerste (University of Hamburg, Germany). The *P{UAS:BACE}* stock was obtained by crossing out the *P{UAS:APP_695_}* transgene from the *P{UAS:APP_695_}; P{UAS:BACE}* parental stock, and is referred to in the text as BACE alone. UAS-Mito-GFP was obtained from Bill Saxton, University of California (Pilling et al., 2006). Bloomington stock *P{UAS-APP_695_-N-myc}, P{UAS:BACE1}* (BL#33798) was used as an additional experimental background and is referred to as APP; BACE (high) in the text. Bloomington stocks Appl {d} w {*} (BL#43632) and w*; *P{UAS-Appl.T}2* (BL#38403) were used to confirm the presence of endogenous Appl-CTFs. Bloomington stock *w^1118^* (BL#3605) was used to generate outcrossed controls and is referred to as *w^−^* in the text. All transgenes are examined in the heterozygous state. All other controls are the transgenic crosses to appropriately control for genetic background, either lacking the *Gal4* driver or *UAS*-linked transgene, as indicated.

For all experiments, ‘induced’ genotypes contain both the *Gal4* driver and the *UAS* responder together in the same genetic background (*elav-Gal4* with *UAS:APP_695_* with *UAS:BACE*). ‘Uninduced’ genotypes refer to both the *Gal4* genotype alone (*elav-Gal4*) and the *UAS* genotype alone (*UAS:APP_695_* with *UAS:BACE, UAS:APP_695_* alone and *UAS:BACE* alone).

### Pharmacological reagents

γ-secretase transition state inhibitor, L-685,458, was from Sigma-Aldrich. 100 nM L-685,458 was used for preparing food vials for AD model flies. Drug or DMSO was added to water and mixed to homogeneity prior to preparing food. DMSO concentration was 0.1% in all cases. Larvae were raised on food containing either drug or DMSO alone for their entire development (starting from embryogenesis). No external yeast was added to this food at any point during the analysis.

### Western blot analysis

For western blot analysis, 15–20 fly heads were collected from indicated genotypes and immediately lysed in RIPA buffer (50 mM Tris, 150 mM NaCl, 1% SDS, 1% NP-40, 0.5% deoxycholate, pH 8.0) containing a cocktail of protease inhibitors [Antipain (100 mM), Aprotinin (2 mg/ml), Benzamide (15 mg/ml), Chymostatin (100 mM), Leupeptin (100 mM), Pepstatin A (1 mM), PMSF (1 mM), Sodium Metabisulfite (0.1 nM)]. These lysates were stored at −80°C. The protein concentration of these fly head lysates was determined using the BCA Protein Assay Kit (Pierce, Inc.). According to the protein concentrations, samples for western blot were prepared using the 4× NuPage LDS sample buffer (Invitrogen, Inc.) containing 0.2% BME (β-Mercaptoethanol, Sigma-Aldrich). Equal amounts of protein were loaded onto each well of NuPAGE 4–12% Bis Tris Gel. From the gel, the proteins were transferred onto 0.25 μm PVDF (Immobilon FL) membrane (Millipore) using a semi-dry transfer apparatus. Blots were probed with the indicated antibodies and imaged using Odyssey Infrared Imaging system (LI-COR Biosciences).

### Antibodies and immunohistochemistry

Antibodies utilized for western blot analysis were C1/6.1 monoclonal antibody recognizing the C-terminus of APP, kindly provided by Paul Mathews (NYU, New York), 3D5 monoclonal antibody recognizing the catalytic domain, residue 46–460 of BACE, and a monoclonal anti-β-actin antibody (A5441, Sigma-Aldrich). The following mouse monoclonal antibodies from the Developmental Studies Hybridoma Bank were used for immunohistochemistry: anti-Brp (nc82, 1:100), anti-DLG (4F3, 1:10), anti-dCSP (1:3000). F-actin was labeled using TRITC-conjugated Phalloidin at 1:200 (Sigma-Aldrich). Neuronal structures were labeled using fluorescein-conjugated HRP at 1:50 (Jackson ImmunoResearch Labs). HRP-Cy5 was used for staining neuronal structure with Mito-GFP larvae (Jackson ImmunoResearch Labs, 1:10). Secondary antibody used for western blot was goat anti-mouse IR Dye 680 (926–3200; Li-Cor Inc.) and for immunohistochemistry was goat anti-mouse Cy5 (# 115-176-072, 1:100) from Jackson ImmunoResearch. The 6/7 NMJ of abdominal hemisegments A3 were used for all studies.

Wandering third instar larvae of both sexes were dissected and fillet preparations were pinned down in Sylgard lined Petri dishes. The larvae were dissected in PBS and fixed in 4% paraformaldehyde for 25 minutes. Larvae were then washed with PBS containing 0.1% Triton X-100 (PBT), then permeabilized with PBS containing 0.5% Triton X-100. Larval body walls were then incubated overnight in primary antibody diluted as indicated in 1% normal goat serum (NGS) in PBT at 4°C, followed by two washes in PBT, overnight incubation in secondary antibody diluted in 1% NGS in PBT at 4°C, a wash with PBT and staining with HRP and Phalloidin for 45 minutes. Larval body walls were mounted in Vectashield (Vector Labs, H-1000). All fluorescent imaging was done using an Olympus FluoView FV1000 laser scanning confocal microscope.

### Behavioral testing

For all behavioral studies, wandering third instar larvae were collected and rinsed briefly with PBS to remove residual food medium. The larvae were then placed in a 4% agar-coated plastic Petri dish. Tests were performed in a separate room maintained at 25°C and 50% humidity, in which phototactic and geotactic cues were eliminated by uniform lighting and flat agar surfaces.

For larval contraction assay, a larva was placed individually at the center of the Petri dish. For each assay, the larva was allowed to move freely for several seconds before the analyses to adjust to the new environment. The number of full body wall contractions (BWC) (forward or backward) that occurred in a 30 second period was counted and converted to BWC/minute. Three consecutive trials were performed for each larva, and these were averaged to produce a single data point. For each genotype, 40–50 larvae were examined. The BWC for all larvae were measured manually using a Leica Mz 125 stereomicroscope.

For larval crawling assay, three larvae of a given genotype were placed on a clean 4% agar-coated plastic Petri dish and allowed to acclimate to the new environment. Larval crawling was digitally recorded using a Sony DCR-SR47 Handycam with Carl Zeiss optics for 5 minutes. Subsequent digital video analysis was quantified for distance and velocity using iMovies software (Apple) and ImageJ plugin ‘Manual tracking’.

### Data acquisition

All images were captured at constant confocal gain settings and at 600× magnification. Images were acquired as a *z*-stack and then rendered as a maximum projection. The total number of boutons and branches were acquired from muscles 6 and 7 in hemisegment A3 of all larval fillets. The Brp-positive puncta were quantified using Image-based Tool for Counting Nuclei (ITCN) plug-in for ImageJ (NIH) with width set to 7, minimum distance to 3.5 and threshold set to 3. Immunofluorescence reactivity for all other synaptic proteins was quantified using ImageJ (NIH) by measuring the mean fluorescence intensity of the NMJ normalized with the mean non-NMJ background, the intensities were further normalized to *Gal4* outcross control. Fluorescence for Mito-GFP was also quantified similarly. Bouton size for 1s and 1b bouton comparisons were measured in ImageJ.

### Statistical analysis

All statistical analyses were performed on SPSS version 20. To determine the significance between multiple different genotypes, a one-way ANOVA analysis was performed with Tukey post-hoc or Games-Howell analysis. Genotype is the independent variable. An unpaired Student’s *t*-test was performed between two groups of different treatments. Significance was determined at the 95% confidence interval.

## Supplementary Material

Supplementary Material
