# Supplementary material for: Synaptic abnormalities in a Drosophila model of Alzheimer’s disease
Source: Dis Model Mech. 2014 Jan 30;7(3):373–85. doi: 10.1242/dmm.012104 (PMC3944497; doi:10.1242/dmm.012104)
Supplement: Supplementary Material [file supp_7_3_373__index.html]

Synaptic abnormalities in a Drosophila model of Alzheimer’s disease — Supplementary Material 

# Synaptic abnormalities in a *Drosophila* model of Alzheimer’s disease

## DMM012104 Supplementary Material

**Files in this Data Supplement:**

- **Supplementary Material**
