# Supplementary material for: Synaptic abnormalities in a Drosophila model of Alzheimer’s disease
Source: Dis Model Mech. 2014 Jan 30;7(3):373–85. doi: 10.1242/dmm.012104 (PMC3944497; doi:10.1242/dmm.012104)
Supplement: Supplementary Material [file supp_7.3.373_DMM012104.pdf]

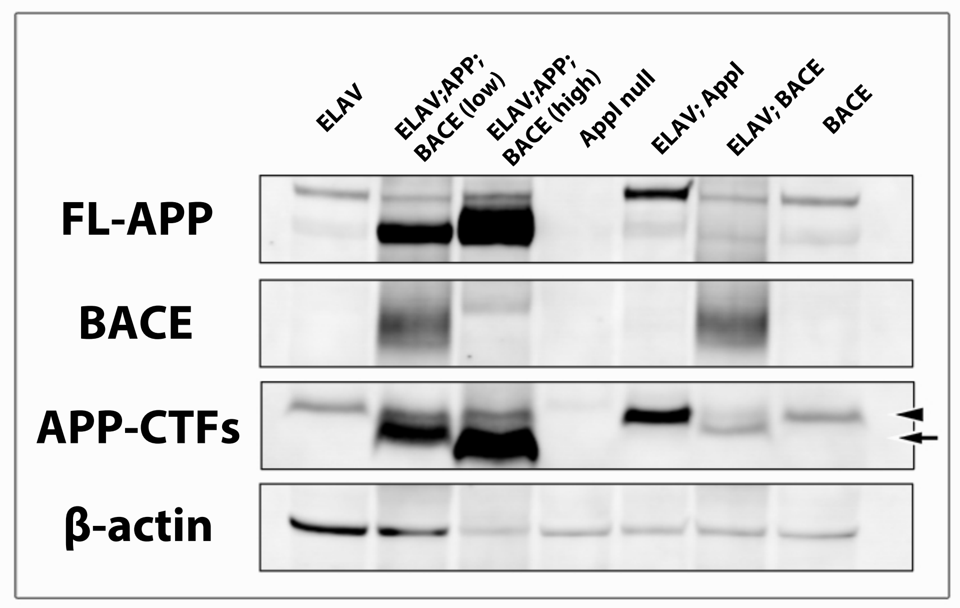

Supplemental Figure 1: Western Blot demonstrating presence of endogenous Appl-CFTs

Western blot analysis of human APP, human BACE, fly Appl and β-actin is detected in fly head lysates. Fly lines over-expressing endogenous Appl and Appl null mutant lines are tested to verify the presence of Appl-CTFs. Lane 1; *elav; +; +* heterozygous flies. Lane 2: *elav; APP; BACE* (low) heterozygous flies. Lane 3: *elav; APP; BACE* (high) heterozygous flies. Lane 4: *Appl* null mutant flies. Lane 5: *elav; Appl;* + heterozygous flies. Lane 6: *elav;* +*; BACE/*+ heterozygous flies. Lane 7: +; +; *BACE/*+ heterozygous flies. FL-APP (full length APP, ~110kD), APP-CTFs (C-terminal fragments, ~10-12 kD) and Appl-CTFs (~15 kD) were detected by C1/6.1. BACE (~70kD) was detected by 3D5 antibody. Arrow indicates β-CTFs in lanes 2 and 3. Arrowhead indicates Appl-CTFs in lanes 1-3, 4-6. β-actin antibody was utilized for loading controls.

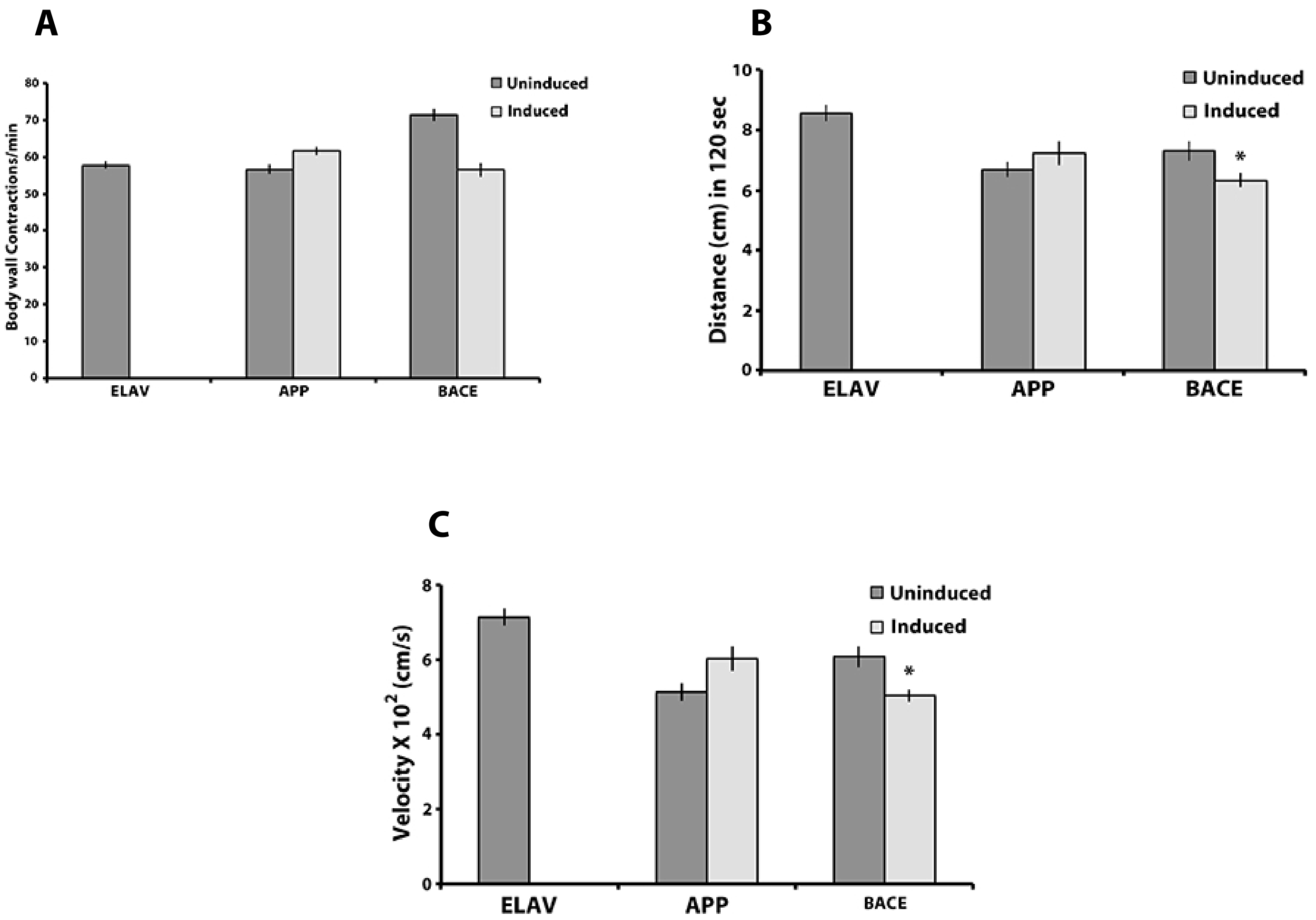

Supplemental Figure 2: Effect of expressing APP alone and BACE alone on behavioral phenotypes of Drosophila 3rd instar larvae.

A) Number of body wall contraction per minute of third instar larvae under induced (*Gal4* with *UAS*) and uninduced (*Gal4* or *UAS* alone) conditions (n>30). **B)** Distance crawled by larvae in 120 sec (n>30). **C)** Crawling velocity in cm/sec for the third instar larvae under examination (n>30). Treatments are indicated. * indicates p-value< 0.05. Error bar represents standard error in each case.

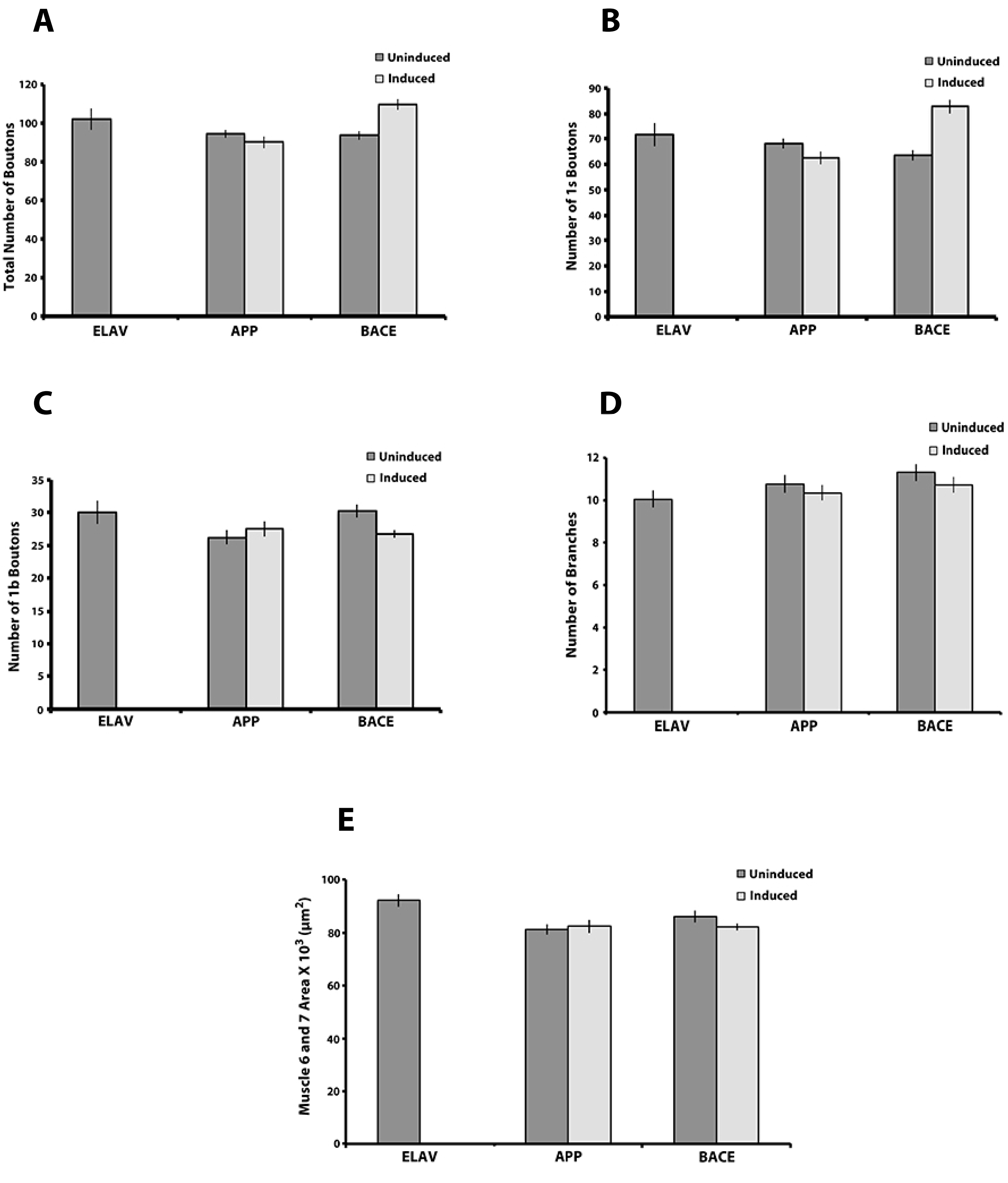

Supplemental Figure 3: Expressing APP alone and BACE alone does not alters synapse formation.

Histogram depicts quantitative analysis of bouton and branch number on muscles 6 and 7 at abdominal segment 3 of larvae expressing APP alone or BACE alone under induced (*Gal4* with *UAS*) and uninduced (*Gal4* or *UAS* alone), where **A)** Total number of boutons, **B)** Number of 1s type boutons, **C)** Number of 1b type boutons, **D)** Total number of branches, **E)** Average muscle 6 and7 area. Treatments are indicated. Analysis represents n > 15. * indicates p-value< 0.05. Error bar represents standard error in each case.

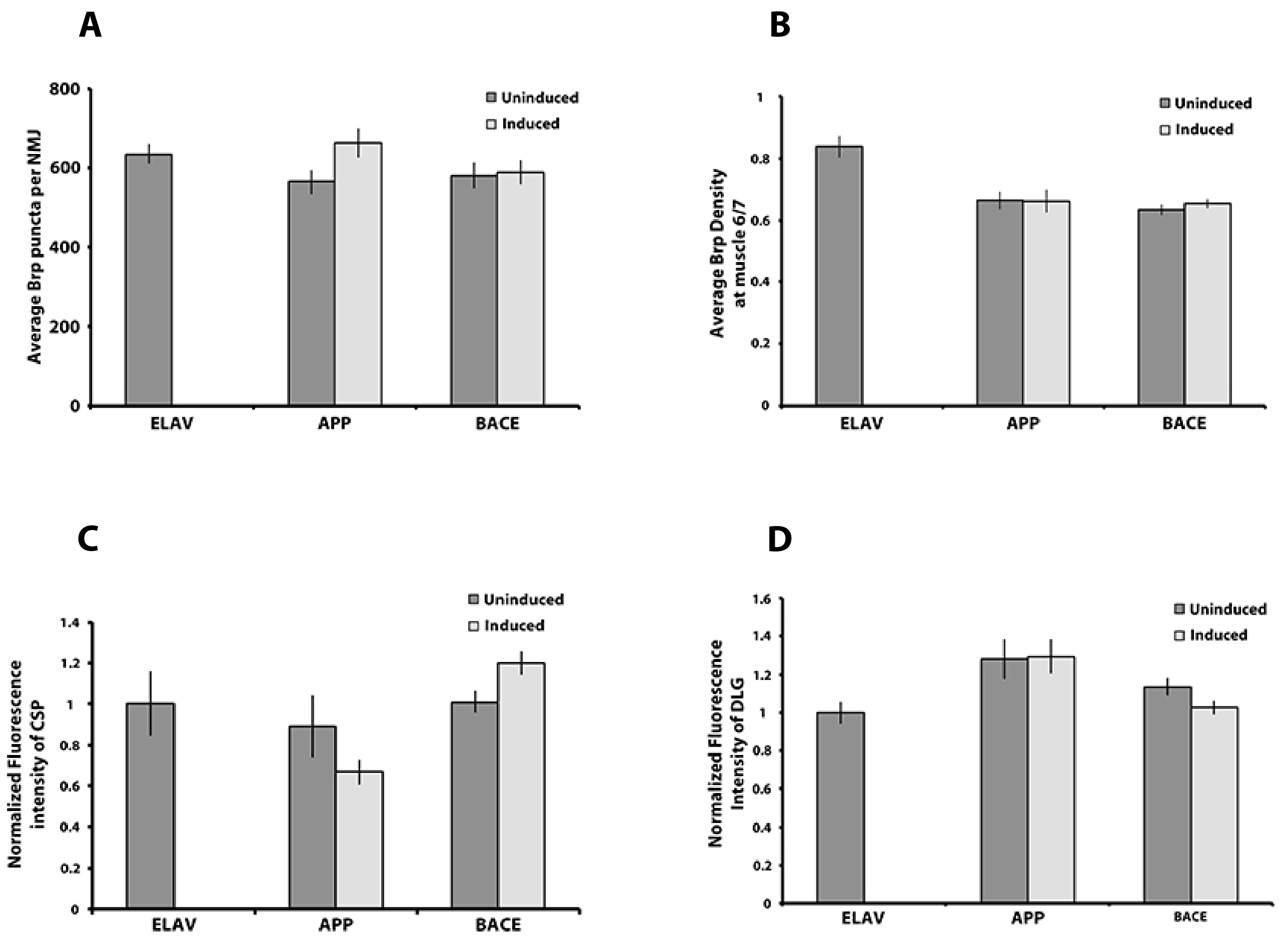

Supplemental Figure 4: Expressing APP alone and BACE alone has no effect on pre- and post-synaptic proteins.

Histogram depicts quantitative analysis pre- and post-synaptic proteins present at the synapse formed on muscles 6 and 7 at abdominal segment 3 of larvae expressing APP alone or BACE alone under induced (*Gal4* with *UAS*) and uninduced (*Gal4* or *UAS* alone), where **A)** Average number of active zones per synapse, **B)** Average Brp density, **C)** Normalized Fluorescence intensity of CSP, and **D)** Normalized Fluorescence intensity of DLG. Treatments are indicated. Analysis represents n = 10-14. * indicates p-value< 0.05. Error bar represents standard error in each case.

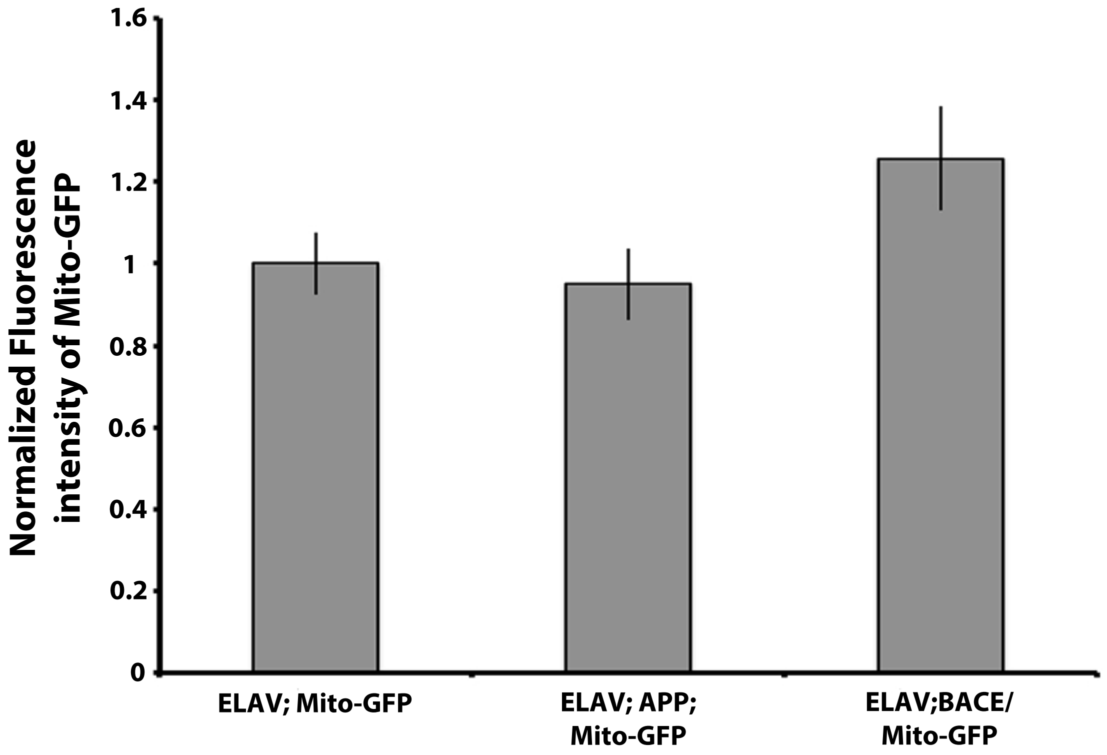

Supplemental Figure 5: Expressing APP alone and BACE alone has no effect on mitochondrial localization in the motor neurons.

Histogram depicts quantitative analysis of normalized fluorescence intensity of Mito-GFP upon expressing APP alone or BACE alone under induced (*Gal4* with *UAS*) and uninduced (*Gal4* or *UAS* alone). Treatment indicated. Analysis represent n= 10-14. * indicates p-value< 0.05. Error bar represents standard error in each case.
